# Antigen-Presenting Human γδ T Cells Promote Intestinal CD4^+^ T Cell Expression of IL-22 and Mucosal Release of Calprotectin

**DOI:** 10.4049/jimmunol.1700003

**Published:** 2017-03-22

**Authors:** Christopher J. Tyler, Neil E. McCarthy, James O. Lindsay, Andrew J. Stagg, Bernhard Moser, Matthias Eberl

**Affiliations:** * Division of Infection and Immunity, School of Medicine, Cardiff University, Cardiff CF14 4XN, United Kingdom;; † Centre for Immunobiology, The Blizard Institute, Barts and The London School of Medicine and Dentistry, Queen Mary University of London, London E1 2AT, United Kingdom;; ‡ Department of Gastroenterology, The Royal London Hospital, Barts Health NHS Trust, London E1 1BB, United Kingdom; and; § Systems Immunity Research Institute, Cardiff University, Cardiff CF14 4XN, United Kingdom

## Abstract

The cytokine IL-22 plays a critical role in mucosal barrier defense, but the mechanisms that promote IL-22 expression in the human intestine remain poorly understood. As human microbe–responsive Vγ9/Vδ2 T cells are abundant in the gut and recognize microbiota-associated metabolites, we assessed their potential to induce IL-22 expression by intestinal CD4^+^ T cells. Vγ9/Vδ2 T cells with characteristics of APCs were generated from human blood and intestinal organ cultures, then cocultured with naive and memory CD4^+^ T cells obtained from human blood or the colon. The potency of blood and intestinal γδ T-APCs was compared with that of monocytes and dendritic cells, by assessing CD4^+^ T cell phenotypes and proliferation as well as cytokine and transcription factor profiles. Vγ9/Vδ2 T cells in human blood, colon, and terminal ileum acquired APC functions upon microbial activation in the presence of microenvironmental signals including IL-15, and were capable of polarizing both blood and colonic CD4^+^ T cells toward distinct effector fates. Unlike monocytes or dendritic cells, gut-homing γδ T-APCs employed an IL-6 independent mechanism to stimulate CD4^+^ T cell expression of IL-22 without upregulating IL-17. In human intestinal organ cultures, microbial activation of Vγ9/Vδ2 T cells promoted mucosal secretion of IL-22 and ICOSL/TNF-α–dependent release of the IL-22 inducible antimicrobial protein calprotectin without modulating IL-17 expression. In conclusion, human γδ T-APCs stimulate CD4^+^ T cell responses distinct from those induced by myeloid APCs to promote local barrier defense via mucosal release of IL-22 and calprotectin. Targeting of γδ T-APC functions may lead to the development of novel gut-directed immunotherapies and vaccines.

## Introduction

Effective host protection against pathogens requires dynamic cross-talk between leukocytes and non-immune cells at epithelial barrier sites including the skin, lung, and intestine, as well as continuous interaction with the commensal microbiota that populate these tissues (1, 2). A key regulator of epithelial integrity and immunity is the cytokine IL-22, which induces secretion of antimicrobial peptides, acute phase proteins and mucins, and drives neutrophil recruitment via production of chemokines (3). The multiple effects of IL-22 mediate epithelial barrier protection in the steady state but can also induce tissue pathology when dysregulated; hence this cytokine has been implicated in inflammatory disorders of epithelial surfaces including psoriasis and inflammatory bowel disease (IBD) (4–6).

Gut-resident innate lymphoid cells (ILCs) are major producers of IL-22 in the mouse intestine (7), but there is a conspicuous reduction in IL-22–producing ILC numbers toward the distal end of the digestive tract, suggesting that other cell types may complement the role of IL-22^+^ ILCs (8, 9). Indeed, the IL-22^+^ ILC population may be functionally redundant in the human gut provided that CD4^+^ T cells, which are prominent sources of IL-22 during intestinal inflammation, are present (10, 11). The immunological mechanisms that induce IL-22 expression in mucosal T cells are poorly understood. IL-22 is typically coexpressed with IFN-γ and/or IL-17 by cells belonging to the Th1 and Th17 lineages, respectively. However, growing evidence also suggests the existence of a distinct Th22 lineage that expresses IL-22 without IL-17 or IFN-γ (12–15). Indeed, human skin–derived Langerhans cells have been reported to induce a distinct population of IL-22^+^ CD4^+^ T cells that lack IL-17, although the underlying molecular mechanism was not defined (14). Similarly, circulating plasmacytoid dendritic cells (DCs) release soluble factors including IL-6 and TNF-α, which stimulate skin-homing CD4^+^ T cells to express IL-22 but not IL-17 (12). Intestinal DCs have also been described to induce IL-22 expression in CD4^+^ T cells, but only in conjunction with other cytokines including IFN-γ, IL-17, and IL-10. The pathways underpinning the specific induction of IL-22^+^ IL-17^−^ CD4^+^ T cells in the human gut remains unknown (16).

Myeloid APCs may not be the only Ag-presenting populations in the intestine that can modulate local CD4^+^ T cell responses. Indeed, microbe-responsive Vγ9/Vδ2 T cells in the human gut express APC markers, influence colonic CD4^+^ T cell function (17), and may contribute to the pathology of IBD (18). Whereas they are absent in rodents, Vγ9/Vδ2 T cells typically represent 1–5% of total T cells in human blood and tissues including the gut (17, 18). Intriguingly, Vγ9/Vδ2 T cells readily acquire APC characteristics in vitro, induce naive and memory CD4^+^ and CD8^+^ T cell responses (19, 20), and thus possess considerable potential for immunotherapeutic applications. However, little is known about the capacity of such γδ T-APCs to polarize CD4^+^ T cell responses, especially in anatomical compartments other than blood.

In this report, we demonstrate that human microbe–responsive Vγ9/Vδ2 T cells readily acquire gut-homing and Ag-presenting functions, and stimulate CD4^+^ T cell responses distinct from those induced by monocytes or DCs. Unlike myeloid APCs, blood and intestinal γδ T-APCs failed to promote IL-17 but were capable of potent IL-22 induction in naive and memory CD4^+^ T cells via a costimulatory pathway that required inducible T-cell costimulator ligand (ICOSL) and TNF-α but not IL-6. Selective induction of IL-22 responses in human intestinal CD4^+^ T cells without parallel upregulation of IL-17 is likely to promote barrier integrity and mediate host protection against pathological inflammation (21), consistent with a critical role for γδ T cells in the immunosurveillance of peripheral tissues (22).

## Materials and Methods

### Ethical approval

Recruitment of patients and healthy volunteers was conducted according to the principles expressed in the Declaration of Helsinki and approved under reference numbers 05/Q0405/71, Harrow Research Ethics Committee; 10/H0704/74, East London Research Ethics Committee 2; P/01/023, East London and City Health Authority Research Ethics Committee; and 08/WSE04/17, South East Wales Local Ethics Committee. All individuals provided written informed consent prior to inclusion in the study.

### Media, reagents, and Abs

For T cell cultures, RPMI 1640 was supplemented with 10% FCS, 50 μg/ml penicillin/streptomycin, 2 mM l-glutamine, 1% sodium pyruvate, and 100 μM non-essential amino acids (Life Technologies). For tissue samples, Dutch-modified RPMI 1640 medium was supplemented with 10% FCS, 2 mM l-glutamine, 100 U/ml penicillin, 100 μg/ml streptomycin, and 25 μg/ml gentamicin (Sigma). Synthetic (*E*)-4-hydroxy-3-methyl-but-2-enyl pyrophosphate (HMB-PP) was purchased from Echelon Biosciences. Toxic shock syndrome toxin-1 (TSST-1) was purchased from Toxin Technology; purified protein derivative (PPD) was from Statens Serum Institut, Copenhagen, Denmark. *Salmonella abortus equi* LPS, peptidoglycan (PGN), PMA, ionomycin, and brefeldin A were purchased from Sigma. CFSE was purchased from Life Technologies. Recombinant IL-1β, IL-4, IL-6, IL-12, IL-15, IL-23, GM-CSF, and TNF-α were purchased from Miltenyi; IL-7 from Peprotech; and TGF-β from BD Biosciences. Recombinant IL-2 (Proleukin) was from Chiron; IL-21 was from Zymogenetics.

For surface phenotyping, anti-CD4:APC-H7 (RPA-T4), anti-CD45RA:APC (HI100), anti-CD83:PE-Cy7 (HB15c), anti-TCR-Vδ2:PE (B6.1), and anti-HLA-DR:APC-H7 (L243) from BD Biosciences; anti-CD40:PE (mAB89) and anti-TCR-Vγ9:PE-Cy5 (Immu360) from Beckman Coulter; anti-CD25:APC (BC96), anti-CD70:FITC (113-16) and anti-CD80:FITC (2D10.4) from eBioscience; and anti-CD3:BV421 (UCHT-1), anti-CD14:BV421 (M5E2), anti-CD86:APC (IT2.2), anti-ICOSL:PE (2D3), anti-CCR7:PE-Cy7 (G043H7), anti-CCR9:AF647 (L053E8), anti-CLA:FITC (HECA-452) and anti-integrin β7 (FIB504) from BioLegend were used, together with appropriate isotype controls. Intracellular cytokines were detected using anti-IFN-γ:BV421 (4S.B3; BioLegend), anti-IL-17A:APC (eBio64DEC17; eBioscience), and anti-IL-22:PE-Cy7 (22URTI; eBioscience).

Blocking reagents used were anti-IFN-γ (B27), anti-IL-4 (8D4-8), and anti-IL-6 (MQ2-13A5) from BioLegend; and soluble TNF-α receptor (sTNFR) p75-IgG1 fusion protein (etanercept/Enbrel; Amgen). Agonistic reagents included anti-CD3 (OKT3), anti-CD28 (CD28.2), and anti-ICOS (ISA-3) from eBioscience. Soluble CD70 (sCD70) was provided by Jannie Borst, the Netherlands Cancer Institute.

### Tissue samples

Biopsies of colonic mucosa were obtained from patients undergoing colonoscopy for cancer screening or investigation of rectal bleeding but with no significant findings. Additional mucosal tissue (terminal ileum and colon) was obtained from patients undergoing surgical resection for colorectal cancer or for non-inflammatory intestinal motility disorders. Endoscopic biopsies or equivalently sized pieces of resected intestinal tissue were washed in calcium- and magnesium-free HBSS containing 1 mM DTT (Sigma) for 15 min to remove mucus and feces, followed by incubation in 1 mM EDTA for 1 h under constant shaking to remove the epithelium. Mucosal tissue pieces were then transferred into 24-well plates for organ cultures in complete tissue medium containing 30 U/ml IL-2 and 20 ng/ml IL-15, in the presence or absence of 10 nM HMB-PP. After 3 d, intact tissues were discarded and the egressed leukocytes seeded into 96-well, round-bottom plates for a further 4 d. At the end of the culture period, intestinal Vδ2^+^ T cells and CD4^+^ T cells were each sorted to >99.2% purity.

### Cell isolation from blood and APC generation

PBMC were isolated from heparinized venous blood of healthy donors or from blood bags supplied by the Welsh Blood Service (Velindre National Health Service Trust) using Lymphoprep (Axis-Shield). CD14^+^ monocytes (>99% purity) were purified from PBMC using anti-CD14 microbeads (Miltenyi). Immature DCs (iDCs) were derived from monocytes over 5–6 d in the presence of 50 ng/ml GM-CSF and 50 ng/ml IL-4. Maturation of iDCs into mature DCs (mDCs), and activation of freshly isolated monocytes was achieved via stimulation for 24 h with 100 ng/ml LPS or 1 μg/ml PGN. Vγ9^+^ T cells and Vδ2^+^ T cells (each >99% purity) were isolated using anti-TCR-Vγ9:PECy5 (Beckman Coulter) or anti-TCR-Vδ2:PE mAbs (BD Biosciences), combined with anti-PE microbeads (Miltenyi). γδ T-APCs were generated by coculture of purified blood Vγ9/Vδ2 T cells with irradiated monocytes (50 Gy) at a 10:1 ratio, in the presence of 10 nM HMB-PP with or without 100 U/ml IL-2 or 20 ng/ml IL-7, IL-15, or IL-21. γδ T-APCs were cultured for 3 d and further purified either by positive selection or cell sorting to purities >99.2%. Bulk CD4^+^ T cells (>95% purity) were isolated from PBMC via negative selection using the CD4^+^ T cell Isolation Kit (Miltenyi). For isolation of naive and memory subsets, bulk CD4^+^ T cells were labeled with anti-CD4, anti-CD45RA, and anti-CCR7 mAbs prior to sorting using a FACSAria II (BD Biosciences) to obtain naive (CD4^+^ CD45RA^+^ CCR7^+^) and memory (CD4^+^ CD45RA^−^ CCR7^−^) populations of >99.4% purity.

### Generation of polarized T helper subsets

Naive CD4^+^ T cells were polarized in anti-CD3 coated flat-bottom 96-well plates (5 μg/ml for Th1 cells, 2.5 μg/ml for all other conditions) in the presence of 1 μg/ml anti-CD28 together with the following reagents: Th1, 100 U/ml IL-2, 20 ng/ml IL-12, and 10 μg/ml anti-IL-4; Th2, 20 ng/ml IL-4 and 10 μg/ml anti-IFN-γ; Th17, 20 ng/ml IL-1β, 50 ng/ml IL-6, 2 ng/ml TGF-β, and 10 μg/ml each of anti-IL-4 and anti-IFN-γ; and Th22, 50 ng/ml IL-6, 20 ng/ml TNF-α, and 10 μg/ml each of anti-IL-4 and anti-IFN-γ.

### APC assays

For MLRs, APCs and allogeneic CD4^+^ T cells were cocultured at a 1:10 ratio. γδ T-APCs were irradiated at 12 Gy prior to coculture with responder cells. For Ag-restricted responses, APCs were pulsed with 1 ng/ml TSST-1 for 1 h and washed three times prior to coculture with autologous CD4^+^ T cells. To assess CD4^+^ T cell responses to complex Ag preparations, APCs were cultured with 1 μg/ml PPD for the final 24 h of APC generation and washed three times prior to coculture with autologous CD4^+^ T cells. Cocultures were incubated for 5 d for proliferation assays using CFSE-labeled responder cells, or for 9 d for analysis of cytokine and transcription-factor expression. For blocking of costimulatory molecules, APCs were preincubated with neutralizing mAbs for 2–3 h and washed three times, prior to addition of CD4^+^ T cells. For inhibition of APC-derived cytokines, appropriate blocking reagents were added directly to MLR assays.

### Cell migration

Migration assays were performed in 96-well HTS transwell plates with 5 μm pores (Corning) using RPMI 1640 supplemented with 5% human serum albumin and 1 mM HEPES (Sigma) as chemotaxis buffer. Serial dilutions of CCL25 (maximum concentration 1 μg/ml) were added to the lower chambers, and chemotaxis buffer was used as a negative control. A total of 100,000 γδ T-APCs were seeded into the upper chambers. After 3 h, the percentage of cells that had migrated to the lower chamber was assessed using AccuCheck counting beads (Thermo Fisher).

### Flow cytometry

Cells were acquired on an eight-color FACSCanto II (BD Biosciences) and analyzed with FlowJo 10.1 (TreeStar). Anti-mouse Ab reactive beads were used to set compensation (Life Technologies). Single leukocytes were gated based on light scatter characteristics and exclusion of fixable Aqua Dead Cell Stain (Invitrogen), followed by gating based on fluorescence minus one controls. For detection of intracellular cytokines, cells were restimulated with 10 ng/ml PMA and 1 μM ionomycin for 5 h, and cultures were supplemented with 10 μg/ml brefeldin A during the last 4 h of the incubation period.

### Quantitative PCR

CD4^+^ T cell responders were sorted from cocultures with irradiated APCs to purities >99.1%. Total RNA was extracted using the RNeasy Micro Kit (Qiagen), and used to generate cDNA with the SuperScript VILO cDNA Synthesis Kit (Thermo Fisher). Transcripts were quantified by real-time quantitative PCR using the ViiA7 Real-Time PCR System (Thermo Fisher). Predesigned TaqMan Gene Expression Assays and the Taqman Universal Master Mix II (no UNG) were used according to the manufacturer’s instructions: T-box transcription factor 21 (*TBX21*), Hs00203436_m1; retinoid-related orphan receptor-γ (*RORC*), Hs01076112_m1; aryl hydrocarbon receptor (*AHR*), Hs00169233_m1; and *PPIL2*, Hs00204962_m1 (all from Thermo Fisher). All samples were measured in triplicate. Measured mRNA abundance was normalized to *PPIL2* (cyclophilin) using the ExpressionSuite Software (Thermo Fisher), and is presented as arbitrary units.

### ELISA

Cell-free supernatants from resting or stimulated Vγ9/Vδ2 T cell, DC, or monocyte cultures were collected after 3 d incubation. Supernatants from 50,000 polarized CD4^+^ T cells were collected after 24 h incubation with 10 ng/ml PMA and 1 μg/ml ionomycin. Supernatants from intestinal tissue cells were collected after 3 d in culture. Soluble cytokines were detected using conventional ELISA kits for IFN-γ and calprotectin (BioLegend); TNF-α, IL-10, IL-17, IL-22, and IL-23 (eBioscience); and IL-1β, IL-6, and IL-12p70 (R&D Systems). All samples were measured in duplicate on a Dynex MRX II reader.

### Statistical analysis and data presentation

Statistical analyses were performed using GraphPad Prism 6.0 software. Data distributions were analyzed using D’Agostino–Pearson omnibus normality tests. Data were analyzed using two-tailed Student *t* tests for normally distributed data and two-tailed Mann–Whitney *U* tests for non-parametric data. Differences between groups were analyzed using one-way ANOVA with Holm–Sidak’s post tests for multiple comparisons of parametric data, or Kruskal–Wallis tests combined with Dunn post tests for non-parametric data. Matched data were analyzed using paired *t* tests or Wilcoxon matched pairs tests for two groups, or Friedman tests combined with Dunn multiple comparison tests for more than two groups. In the graphs, each data point represents an individual donor; statistically significant differences are *p < 0.05, **p < 0.01, and ***p < 0.001. Horizontal lines display the median and error bars indicate the interquartile range.

## Results

### Human gut γδ T cells polarize naive CD4^+^ T cells toward production of IFN-γ and IL-22

**FIGURE 1.**
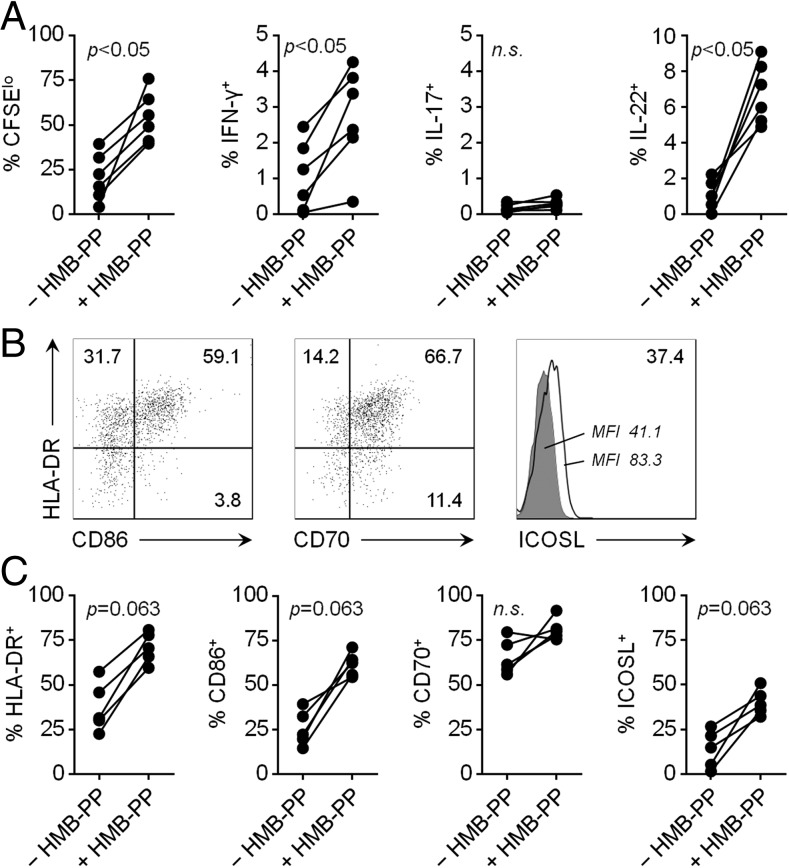
Human mucosal γδ T cells exhibit potent APC activity. (**A**) Blood-derived naive CD4^+^ T cell responses to allogeneic γδ T-APCs from the human colon, shown as CFSE dilution and cytokine expression upon restimulation after 9 d. (**B** and **C**) Expression of APC markers by human gut-derived Vγ9/Vδ2 T cells among total colon cells after 7 d in culture, as gated on live single Vγ9^+^ T cells, compared with unstimulated controls. Data were analyzed using Wilcoxon matched-pairs signed rank tests. FACS plots are representative of ≥4 experiments using HMB-PP stimulated cells from ≥4 donors; numbers indicate percentages together with mean fluorescence intensity (MFI) values for ICOSL expression.

To assess the potential of γδ T cells to exert APC functions in the human gut, colonic organ cultures were either left untreated or stimulated with the microbial Vγ9/Vδ2 T cell ligand HMB-PP, which is produced by the majority of intestinal commensals (23, 24), in the presence of the T cell growth factors IL-2 and IL-15. Egressed Vγ9/Vδ2^+^ γδ T cells were sorted to purity and cocultured with naive CD4^+^ T cells from allogeneic blood donors. Colonic Vγ9/Vδ2 T cells from HMB-PP–stimulated gut tissue displayed an increased ability to induce proliferation and differentiation of naive CD4^+^ T cells toward expression of IFN-γ and IL-22, compared with unstimulated controls (Fig. 1A), whereas only trace numbers of IL-17^+^ CD4^+^ T cells were generated in these cocultures. Similar results were obtained with human ileum (data not shown), demonstrating that Vγ9/Vδ2 T cells in both the small and large intestine exert comparable effects on CD4^+^ T cell responses. These functional data were reflected by the expression of MHC class II and costimulatory molecules commonly associated with APCs (Fig. 1B). Egressed gut Vγ9/Vδ2 T cells already expressed intermediate levels of HLA-DR, CD86, CD70, and ICOSL at baseline, and all bar CD70 were markedly upregulated in response to exogenous HMB-PP (Fig. 1C). These data indicate that intestinal Vγ9/Vδ2 T cells display APC features ex vivo.

### Microbe-responsive γδ T cells acquire a gut-homing APC phenotype upon TCR triggering in a cytokine-dependent manner

**FIGURE 2.**
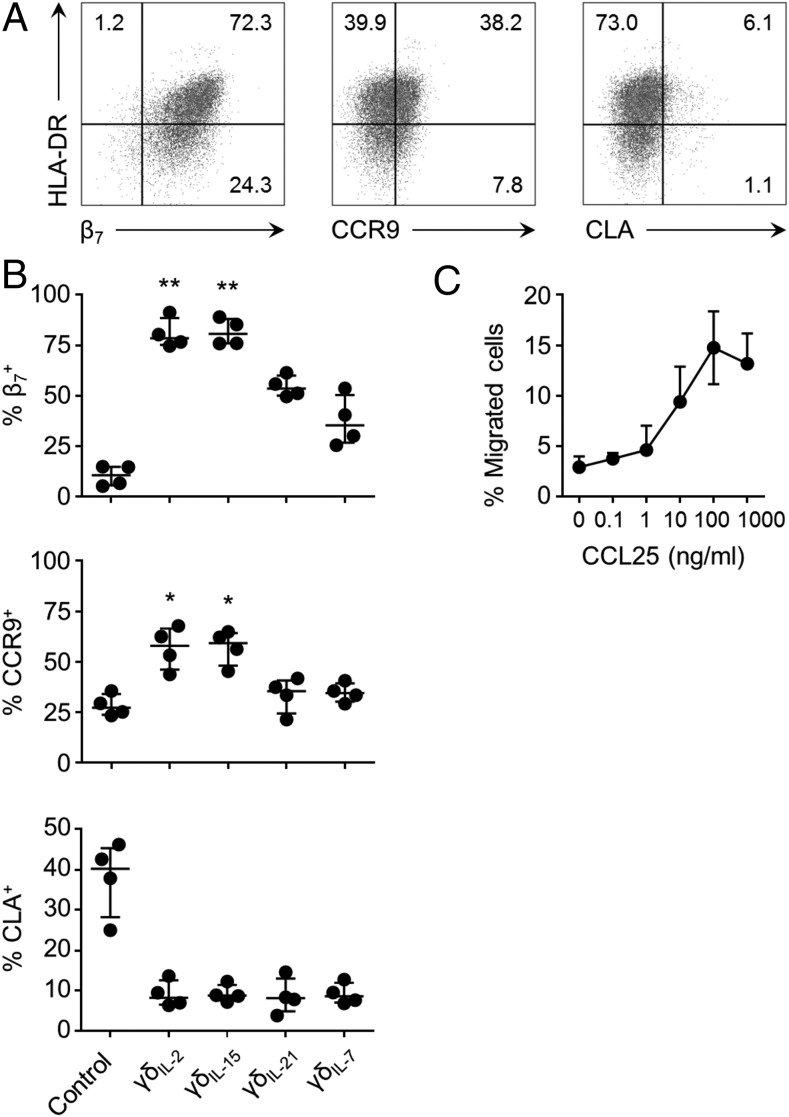
Gut-homing potential of γδ T-APCs. (**A**) Expression of β_7_ integrin, CCR9, and CLA by γδ_IL-15_ T-APCs on day 3 of culture. (**B**) Expression of gut and skin homing markers by γδ T-APCs generated in the presence of the indicated cytokines, as gated on live single Vγ9^+^ T cells. (**C**) Migration of γδ_IL-15_ T-APCs toward CCL25, shown as percentage of total input cells. Data in (B) were analyzed using Kruskal–Wallis tests combined with Dunn multiple comparison tests compared with controls in the absence of APCs. FACS plots are representative of ≥4 experiments using cells from ≥4 donors; numbers indicate percentages. *p < 0.05, **p < 0.01.

In support of a role in the intestine, blood Vγ9/Vδ2 T cells stimulated with HMB-PP in the presence of either IL-2 (γδ_IL-2_ T-APCs) or IL-15 (γδ_IL-15_ T-APCs) expressed β_7_ integrin and the small bowel-homing chemokine receptor CCR9 but only trace levels of the skin-homing cutaneous lymphocyte–associated Ag (CLA) (Fig. 2A, 2B). These data evoke our previous report that upregulation of the α_4_β_7_ heterodimer on blood Vγ9/Vδ2 T cells is accompanied by increased binding to the mucosal addressin MAdCAM-1 as well as decreased CLA expression (17). Whereas IL-2 and IL-15 each supported upregulation of a gut-tropic Vγ9/Vδ2 T cell phenotype, the closely related cytokines IL-7 and IL-21 failed to induce CCR9 (Fig. 2B). Confirming a functional role for CCR9, γδ_IL-15_ T-APCs readily migrated toward its ligand CCL25 (Fig. 2C).

**FIGURE 3.**
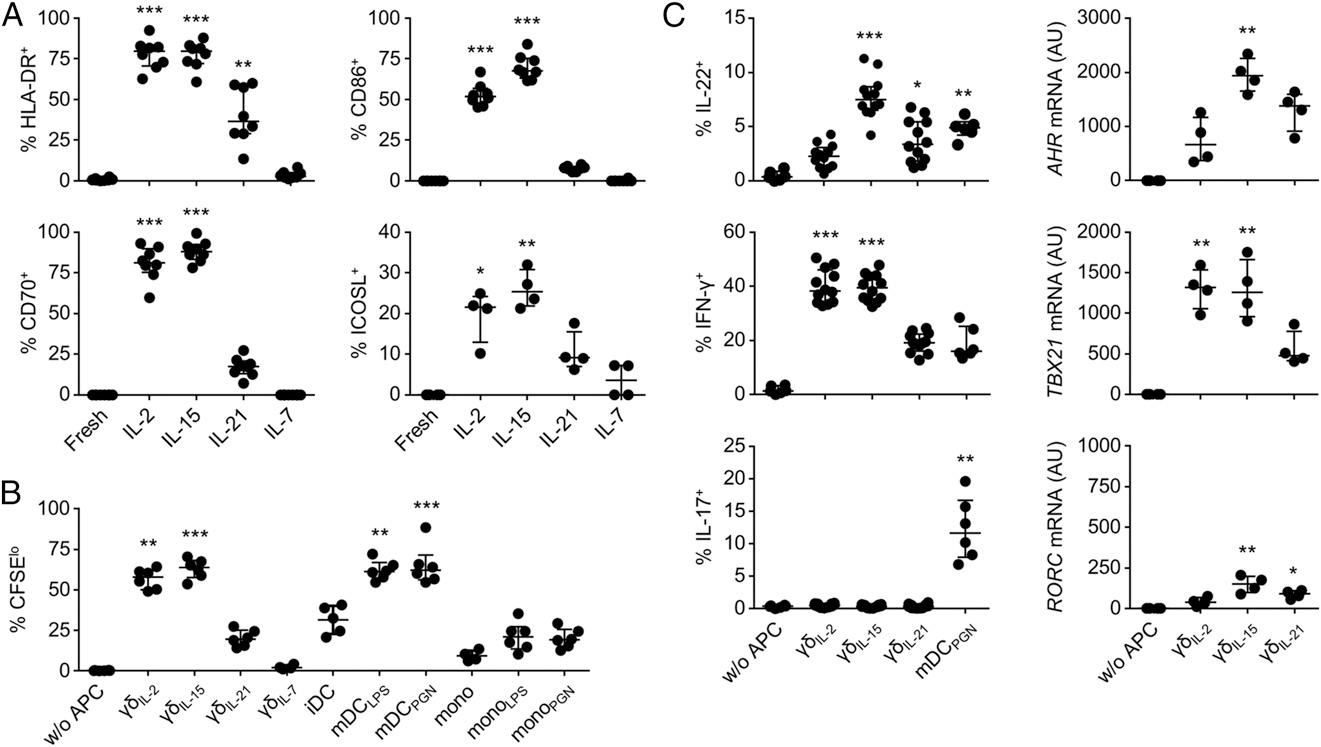
Naive CD4^+^ T cell polarization by γδ T-APCs. (**A**) Expression of APC markers by freshly isolated Vγ9/Vδ2 T cells and by Vγ9/Vδ2 T cells cocultured for 3 d with irradiated autologous monocytes in the presence of HMB-PP with the indicated cytokines, as gated on live single Vγ9^+^ T cells. (**B**) Proliferation of CFSE-labeled naive CD4^+^ T cells in response to allogeneic γδ T-APCs generated under different conditions, compared with iDCs, LPS or PGN-matured DCs (mDC_LPS_, mDC_PGN_), freshly isolated monocytes (mono), and LPS- or PGN-stimulated monocytes (mono_LPS_, mono_PGN_). (**C**) Polarization of naive CD4^+^ T cells by allogeneic γδ T-APCs generated under different conditions, compared with mDC_PGN_, as determined after 9 d upon restimulation. Transcription factors were measured after FACS sorting of polarized CD4^+^ T cells from APC cocultures. Relative expression was determined relative to naive CD4^+^ T cells. Data were analyzed using Kruskal–Wallis tests combined with Dunn multiple comparison tests versus freshly isolated cells (A) or controls in the absence of APCs (C). FACS plots are representative of ≥4 experiments using cells from ≥4 donors. *p < 0.05, **p < 0.01, ***p < 0.001. AU, artificial units.

When combined with TCR triggering, both IL-2 and IL-15 also supported Vγ9/Vδ2 T cell upregulation of HLA-DR, CD86, CD70, and ICOSL (Fig. 3A), as well as expression of CD40, CD80, CD83, and the lymph node–homing receptor CCR7 (Supplemental Fig. 1). In contrast, IL-21 induced only low or intermediate levels of the APC markers tested in this study, and IL-7 treated Vγ9/Vδ2 T cells did not exhibit any discernible APC phenotype, thus serving as a negative control in subsequent functional assays. These findings indicate that IL-2 and IL-15 play crucial and distinct roles in the acquisition of a gut-homing APC phenotype by human Vγ9/Vδ2 T cells.

### γδ T-APCs polarize naive CD4^+^ T cells toward distinct effector phenotypes

When tested in MLRs, γδ T-APCs readily induced naive CD4^+^ T cell proliferation. Both γδ_IL-2_ and γδ_IL-15_ T-APCs drove naive CD4^+^ T cell proliferation with comparable efficiency to that displayed by monocyte-derived DCs matured in the presence of LPS or PGN (Fig. 3B) (25). This effect was abrogated with neutralizing mAbs against CD11a (data not shown), a treatment known to disrupt the immunological synapse. γδ_IL-21_ T-APCs displayed weak APC activity, comparable with that of LPS-/PGN-stimulated monocytes, whereas IL-7 treated Vγ9/Vδ2 T cells failed to induce naive CD4^+^ T cell proliferation, in agreement with their observed lack of HLA-DR expression (Fig. 3B).

When assessing the resulting effector functions of stimulated CD4^+^ T cells, we observed striking outcomes depending on the type of APC used. Unexpectedly, the ability of γδ_IL-15_ T-APCs to induce IL-22^+^ CD4^+^ T cells was far superior to that of DCs, monocytes or any other population of γδ T-APCs tested, as judged by intracellular cytokine staining (Fig. 3C) and ELISA (Supplemental Fig. 2A), pointing toward a unique capacity of γδ_IL-15_ T-APCs to promote IL-22 responses. Accordingly, γδ_IL-15_–stimulated CD4^+^ T cells expressed the highest levels of the Th22 associated transcription factor *AHR*. Both γδ_IL-2_ and γδ_IL-15_ T-APCs also efficiently polarized naive CD4^+^ T cells toward IFN-γ production and expression of the Th1 associated transcription factor *TBX21* (Fig. 3C, Supplemental Fig. 2A). Unlike PGN-treated DCs or monocytes, γδ T-APCs failed to give rise to IL-17^+^ CD4^+^ T cells despite background expression of the Th17 master switch retinoid-related orphan receptor-γ (*RORC*) (Fig. 3C), which in human cells can be expressed independently of IL-17 (25). A substantial proportion of IL-22^+^ CD4^+^ T cells was negative for both IFN-γ and IL-17, thus representing bona fide Th22 cells (Supplemental Fig. 2B). Taken together, these findings demonstrate that γδ T-APC plasticity exerts a profound influence on the polarization of naive CD4^+^ T cells, and that γδ_IL-15_ T-APCs display an unexpected capacity to promote the differentiation of IL-22^+^ IL-17^−^ CD4^+^ T cells.

### γδ_IL-15_ T-APCs enhance IL-22 responses in memory CD4^+^ T cells

**FIGURE 4.**
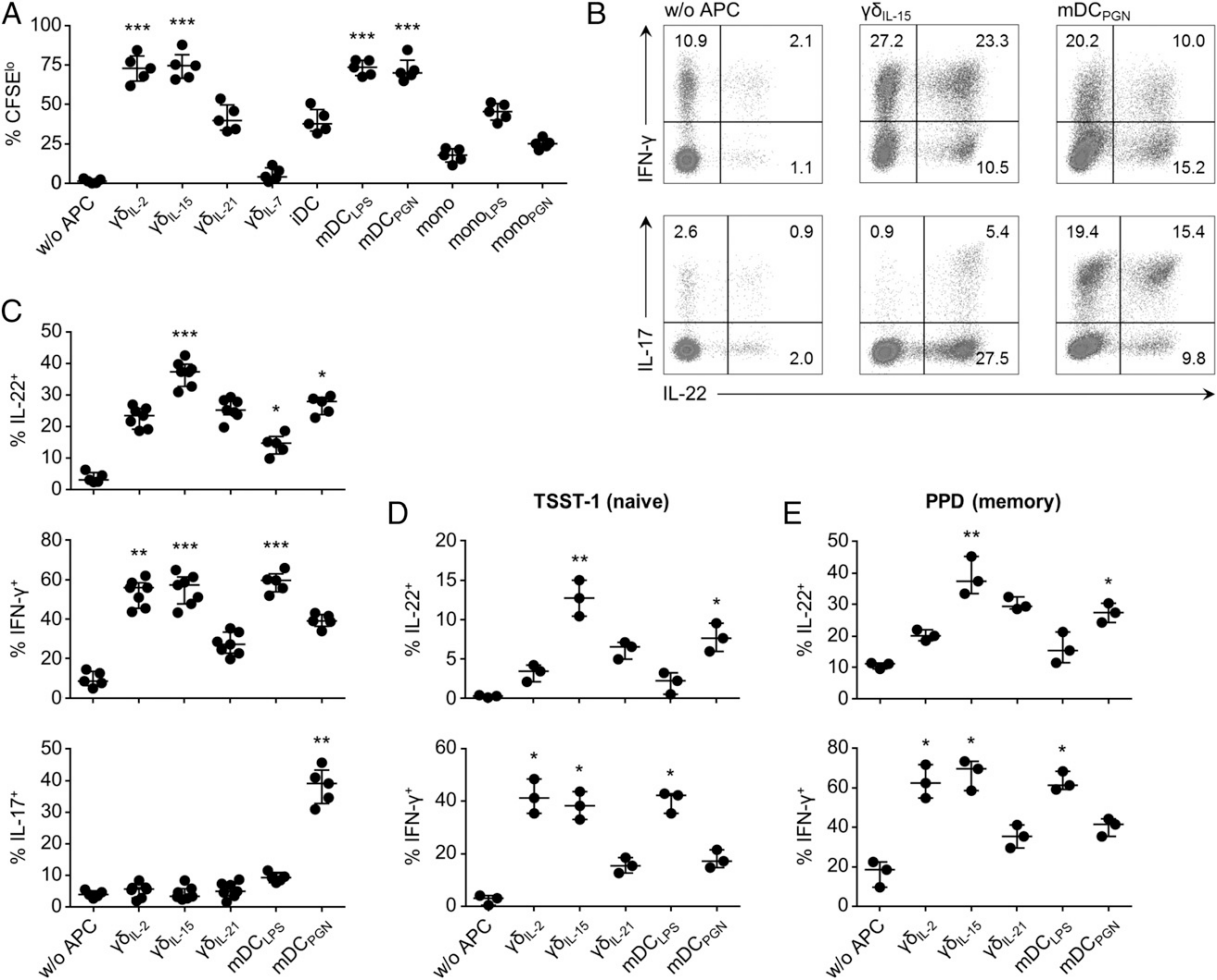
Polarization of memory and Ag-specific CD4^+^ T cells by γδ T-APCs. (**A**) Proliferation of CFSE-labeled memory CD4^+^ T cells in response to allogeneic APCs, displayed as percentage of CFSE^lo^ cells. (**B**) Polarization of memory CD4^+^ T cells by allogeneic γδ_IL-15_ T-APCs or mDC_PGN_, as determined after 9 d upon restimulation. (**C**) Polarization of memory CD4^+^ T cells by allogeneic APCs. (**D**) Polarization of superantigen-specific naive CD4^+^ T cells in response to autologous APCs pulsed with TSST-1, within the Vβ2^+^ CD4^+^ gate. (**E**) Polarization of microbial Ag-specific memory CD4^+^ T cells in response to autologous APCs in the presence of PPD. Data were analyzed using Kruskal–Wallis tests combined with Dunn multiple comparison tests versus controls in the absence of APCs. FACS plots are representative of ≥4 experiments using cells from ≥4 donors. *p < 0.05, **p < 0.01, ***p < 0.001.

We next assessed whether γδ T-APCs could similarly influence the outcome of established effector/memory CD4^+^ T cell responses. As with naive CD4^+^ T cells, both γδ_IL-2_ and γδ_IL-15_ T-APCs induced memory CD4^+^ T cell proliferation with comparable efficiency to that displayed by mDCs (Fig. 4A). Importantly, γδ_IL-15_ T-APCs were the most potent inducers of IL-22 expression, yielding up to 40% IL-22^+^ memory CD4^+^ T cells (Fig. 4B, 4C). In contrast, there was a negligible expansion of IL-17^+^ CD4^+^ T cells in cocultures with γδ T-APCs, as opposed to PGN-treated DCs. Similar results were obtained using autologous responder cells, demonstrating a clear ability of γδ_IL-15_ T-APCs to induce IL-22 responses in autologous TSST-1 specific naive CD4^+^ T cells (Fig. 4D), and in *Mycobacterium tuberculosis* PPD-specific memory CD4^+^ T cells (Fig. 4E). These findings demonstrate that γδ T-APCs can shape Ag-specific adaptive immune responses and induce IL-22 production in both naive and memory CD4^+^ T cells.

### γδ T-APCs polarize CD4^+^ T cells via TNF-α and ICOSL

**FIGURE 5.**
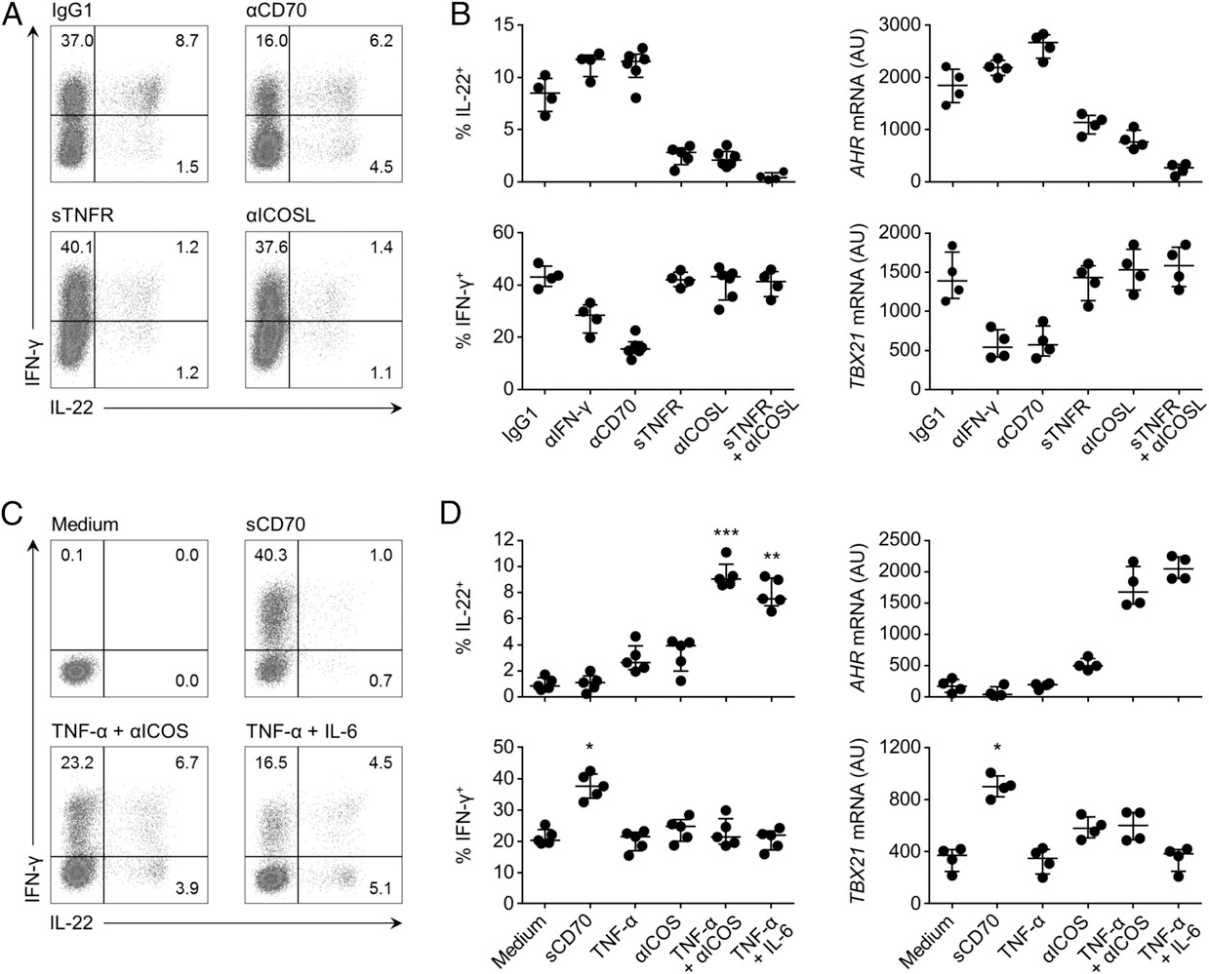
Modulation of naive CD4^+^ T cell responses by polarizing cytokines and costimulatory molecules expressed by γδ T-APCs. (**A** and **B**) Cytokine and transcription factor profiles of naive CD4^+^ T cells cocultured with allogeneic γδ_IL-15_ T-APCs in the absence or presence of neutralizing reagents against IFN-γ, CD70, ICOSL, or sTNFR. (**C** and **D**) Cytokine and transcription factor profiles of naive CD4^+^ T cells stimulated with anti-CD3/CD28 mAbs in the presence of sCD70, TNF-α, IL-6, or agonistic mAbs against ICOS. Data were analyzed using Kruskal–Wallis tests combined with Dunn multiple comparison tests compared with IgG1 (B) or medium controls (D). FACS plots are representative of ≥4 experiments using cells from ≥4 donors. *p < 0.05, **p < 0.01, ***p < 0.001.

We next sought to identify the polarizing signals that facilitate γδ T-APC polarization of T cell responses (Fig. 5). Blocking either CD80 or CD86 reduced naive CD4^+^ T cell proliferation and cytokine production in response to allogeneic γδ_IL-15_ T-APCs (Supplemental Fig. 3A), in agreement with the known role of CD28 signaling in CD4^+^ T cell activation (26). The induction of IL-22 and *AHR* expression in CD4^+^ T cells was selectively impaired in the presence of sTNFR (Fig. 5A, 5B), whereas blocking mAbs against IFN-γ (Fig. 5B) and IL-6 (Supplemental Fig. 3B) exerted no such effect. Consistent with these data, γδ_IL-15_ T-APCs secreted substantial levels of TNF-α but did not produce other DC-associated cytokines such as IL-1β, IL-6, IL-10, IL-12p70, and IL-23 (Supplemental Fig. 4). In addition to the polarizing effect of soluble TNF-α, we observed a crucial contribution of γδ T-APC expressed ICOSL to the induction of IL-22 and *AHR* in naive CD4^+^ T cells (Fig. 5A, 5B). Neutralization of ICOSL did not affect CD4^+^ T cell proliferation and viability (Supplemental Fig. 3A) nor expression of IFN-γ and *TBX21* (Fig. 5B) when assessed in cocultures with γδ_IL-15_ T-APCs, thus demonstrating the specificity of ICOSL signaling for promoting IL-22 expression. Aside from the IL-22 promoting effects of ICOSL and TNF-α, we detected a prominent role for γδ T-APC–derived IFN-γ and CD70 in driving upregulation of IFN-γ and *TBX21* expression (Fig. 5A, 5B).

When assessing the polarizing efficiency of these signals in the absence of APCs using anti-CD3/CD28 mAbs as stimulus, recombinant TNF-α and inducible T-cell costimulator (ICOS) agonists promoted CD4^+^ T cell expression of IL-22 and *AHR* with comparable efficiency to that achieved by supplementation with TNF-α and IL-6 (Fig. 5C, 5D) (12). In contrast, ligation of CD27 on naive CD4^+^ T cells using soluble CD70 (sCD70) led to increased expression of IFN-γ and *TBX21* (Fig. 5C, 5D), consistent with a role for CD27 signaling in Th1 cell differentiation (27). These findings underscore the functional relevance of costimulatory molecule expression by activated Vγ9/Vδ2 T cells and confirm a previously unknown role for ICOS signaling in the induction of human IL-22 responses.

### Gut-homing γδ T-APCs enhance human colonic CD4^+^ T cell responses and promote mucosal release of calprotectin

**FIGURE 6.**
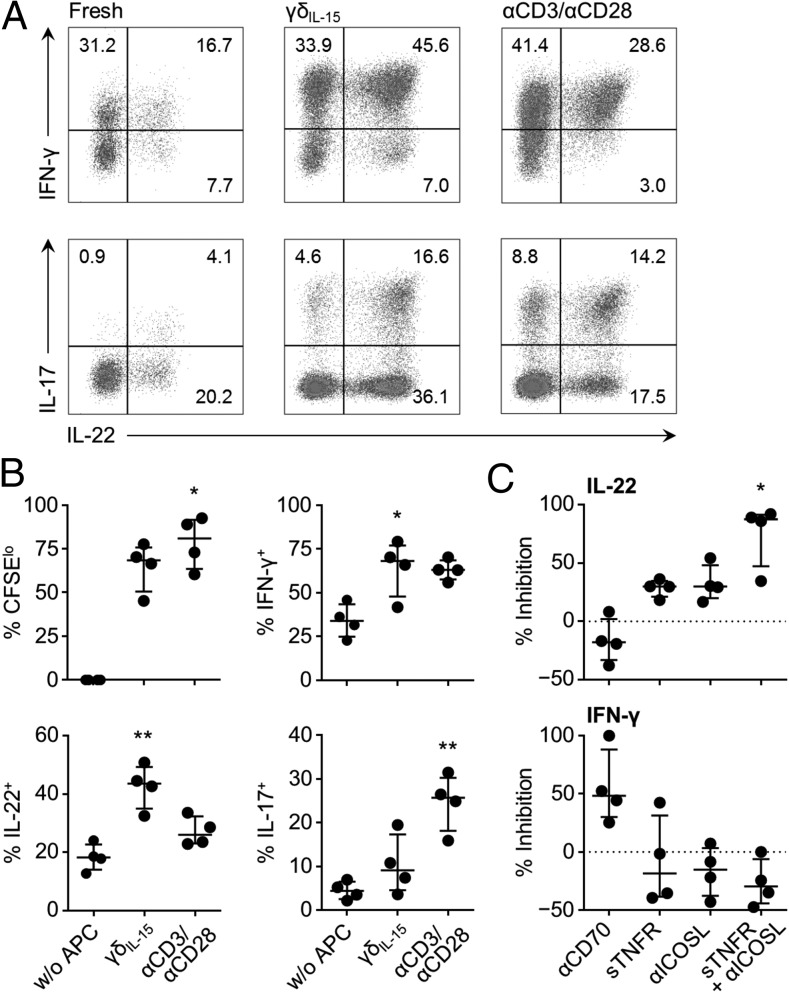
Polarization of human colonic CD4^+^ T cells. (**A**) Intracellular cytokine expression by freshly isolated colonic CD4^+^ T cells (Fresh), and by colonic CD4^+^ T cells cocultured for 9 d with allogeneic γδ_IL-15_ T-APCs or anti-CD3/CD28 mAbs. (**B**) Colonic CD4^+^ T cell responses to allogeneic γδ_IL-15_ T-APCs or anti-CD3/CD28 mAbs. (**C**) Inhibition of the γδ_IL-15_ T-APC–dependent cytokine secretion by colonic CD4^+^ T cells in the presence of the indicated blocking reagents, expressed relative to controls cultured without APCs (100%) and cocultured with allogeneic γδ_IL-15_ T-APCs (0%). Data were analyzed using Kruskal–Wallis tests combined with Dunn multiple comparison tests versus controls in the absence of APCs. FACS plots are representative of ≥4 experiments using cells from ≥4 donors. *p < 0.05, **p < 0.01.

We next tested whether γδ T-APCs are capable of modulating cytokine production by CD4^+^ T cells isolated from human colon, which contains significantly higher frequencies of memory cells than are present in the blood (Fig. 6A). Both anti-CD3/CD28 Abs and allogeneic γδ_IL-15_ T-APCs triggered similar levels of proliferation and IFN-γ production by human colonic CD4^+^ T cells. However, whereas anti-CD3/CD28 stimulation also increased IL-17 production, γδ_IL-15_ T-APCs instead skewed the response toward production of IL-22, which was expressed by up to 50% of all colonic CD4^+^ T cells (Fig. 6B). Combined blockade of TNF-α and ICOSL inhibited IL-22 upregulation by colonic CD4^+^ T cells, whereas neutralizing Abs against CD70 significantly impaired the induction of IFN-γ (Fig. 6C).

**FIGURE 7.**
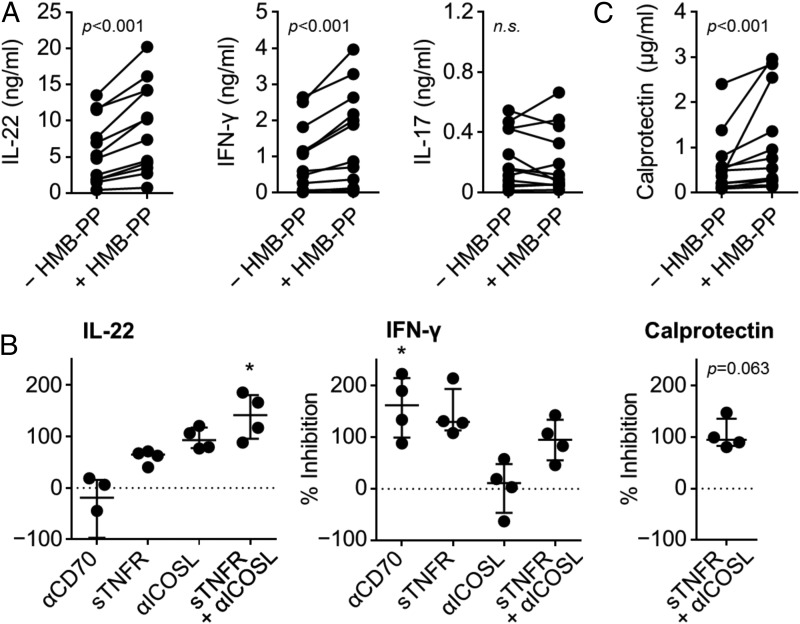
ICOSL-/TNF-α–dependent secretion of IFN-γ, IL-22, and calprotectin by HMB-PP–stimulated human colon. (**A**) Cytokine secretion by total colon biopsy cells cultured for 3 d with IL-2 and IL-15, in the absence or presence of HMB-PP. (**B**) Inhibition of the HMB-PP induced cytokine secretion and calprotectin release from colon organ cultures using the indicated blocking reagents, expressed relative to controls cultured without blocking reagents in the absence (100%) and presence (0%) of HMB-PP. (**C**) Secretion of calprotectin by colonic biopsy cells cultured as in (A), and inhibition of the HMB-PP induced release of calprotectin using sTNFR and anti-ICOSL. Data were analyzed using Wilcoxon matched-pairs signed rank tests (B) or Friedman tests combined with Dunn multiple comparison tests (C) compared with HMB-PP stimulated controls cultured without blocking reagents.

Supernatant concentrations of both IFN-γ and IL-22 were increased in HMB-PP–treated mucosal tissue cultures, whereas IL-17 levels remained unchanged (Fig. 7A). HMB-PP also failed to induce TNF-α, TGF-β, IL-4, or IL-10 secretion (data not shown). Blocking experiments confirmed a major role for TNF-α/ICOSL in driving intestinal IL-22 secretion and demonstrated a significant influence of CD70 on IFN-γ release by total colonic tissue cells (Fig. 7B). Finally, we measured supernatant levels of the antimicrobial protein calprotectin, a heterodimeric complex of the metal ion-binding proteins S100A8 (MRP8) and S100A9 (MRP14), which is produced by epithelial cells in an IL-22–dependent manner, as well as by myeloid lineage cells including granulocytes, monocytes, and tissue macrophages (28, 29). HMB-PP stimulated a significant increase in mucosal tissue release of calprotectin in parallel with increased IL-22 secretion in both colon (Fig. 7C) and ileum organ cultures (data not shown), but not upon neutralization of TNF-α and ICOSL (Fig. 7C). Together, these data reveal that TNF-α and ICOSL expressed by microbe-responsive γδ T cells induce key mediators of epithelial barrier protection and anti-microbial immunity in the human intestine.

## Discussion

This study demonstrates that microbial activation of human Vγ9/Vδ2 T cells in the peripheral blood, colon, and ileum stimulates these cells to act as professional APCs that can efficiently polarize CD4^+^ T cells toward specific effector fates distinct from those induced by monocytes or DCs. Acquisition of γδ T-APC function was potently induced by microbial activation in the presence of the epithelial-derived cytokine IL-15, suggesting a role in local barrier defense via induction of IL-22 and calprotectin release, whereas symbiosis-regulating Th17 responses may instead be mediated by myeloid APCs. Although the unusual potential of human Vγ9/Vδ2 T cells to act as professional APCs for MHC class I– and MHC class II–restricted αβ T cells was discovered more than a decade ago (19), the qualitative nature and physiological relevance of these responses has remained elusive until now, in part due the fact that rodents lack a functional equivalent of microbe-responsive γδ T-APCs (20). Our data now demonstrate that the striking plasticity of effector functions displayed by human Vγ9/Vδ2 T cells (30) can directly influence the cytokine profile of responding CD4^+^ T cells, and that the capacity of γδ T-APCs to prime naive and memory CD4^+^ T cells critically depends on microenvironmental factors present during APC generation. In particular, we identified an unexpected requirement for IL-15 in driving γδ T-APCs to acquire a gut-homing phenotype and the capacity to induce IL-22 production in CD4^+^ T cells. These findings indicate that γδ T-APCs and myeloid APCs provide fundamentally different signals to CD4^+^ T cells.

The physiological relevance of γδ T-APC–induced CD4^+^ T cell responses was evident from our analyses of human intestinal organ cultures, in which Vγ9/Vδ2 T cells were potent inducers of IL-22 expression and promoted the release of calprotectin but not IL-17. Our data demonstrate that human intestinal Vγ9/Vδ2 T cells efficiently upregulate APC features ex vivo, and that this process may readily occur under physiological conditions in vivo where IL-15 is abundantly expressed by the gut epithelium (31, 32) and HMB-PP is produced by the majority of the intestinal microbiota (23). Indeed, while Vγ9/Vδ2 T cells comprise a sizeable fraction of circulating T cells, they are readily recruited to epithelial sites including the skin and intestine, which are subject to microbial challenge (17, 33, 34). Activation of Vγ9/Vδ2 T cells in human intestine may therefore represent an effective mechanism of sensing breaches in the gut barrier and eliciting local protection via IL-22 induction without altering mucosal levels of the prosymbiotic cytokine IL-17 (35), which alters intestinal permeability and exacerbates gut inflammation in patients (36, 37). Our observation that the induction of IL-22 responses in CD4^+^ T cells by γδ T-APCs does not require the inflammatory mediator IL-6, which is a key driver of Th22 differentiation in other settings (12), further suggests a role for γδ T cells in immune surveillance in the healthy gut.

The failure of γδ T-APCs to produce DC-associated polarizing cytokines prompted us to consider alternative signals in driving IL-22 production in CD4^+^ T cells. Classically, CD4^+^ T cell polarization is thought to be predominantly mediated via soluble factors, whereas the influence of costimulatory interactions has been largely overlooked. Our findings identify a novel ICOSL-dependent pathway that polarizes CD4^+^ T cells toward expression of IL-22 and *AHR*, which evokes an earlier report of a role for OX40L in polarizing CD4^+^ T cells toward a follicular T helper cell phenotype (38). This unexpected role for ICOSL in promoting IL-22 expression and calprotectin release from human mucosal tissues has major implications for host protection against microbial infection and inflammation. Because functional IL-22 receptors are expressed by non-immune cells and epithelial IL-15 is ubiquitous not only in the intestine but also in the skin, lung, liver, pancreas, and kidney (4–6), it is likely that our findings apply to the modulation of tissue homeostasis and inflammation at epithelial barriers in multiple organs (6, 13, 39, 40).

IL-22 is a critical mediator of gut barrier defense against bacterial pathogens (41) and underpins therapeutic helminth infection in human colitis (42). In particular, IL-22 triggers the production of antimicrobial mediators including calprotectin, which exerts complex effects on the intestinal microbiota by sequestering essential metal ions (43), inducing epithelial shedding of fucosylated host proteins for metabolism by luminal bacteria (44), and modulating neutrophil functions (45, 46). These effects may radically alter the balance of microbial species that colonize the gut, thus providing effective protection against opportunistic pathogens and chemical-induced colitis (47), but also suppressing commensal species to assist *Salmonella* growth and dissemination from the inflamed gut (3, 48, 49). Accordingly, calprotectin levels in the stool are a reliable indicator of mucosal inflammation and can be used to predict relapse in IBD (50). Our findings therefore uncover new potential targets for therapeutic interventions and may in part explain the beneficial outcomes in patients receiving azathioprine (18).

In summary, we have defined an unexpected role for human microbe–responsive γδ T cells in immune surveillance of human tissues by instructing the cytokine profile of newly primed CD4^+^ T cells and modulating pre-existing memory CD4^+^ T cell function in blood and intestine. The mechanisms described in this study are likely to contribute to epithelial immunosurveillance and barrier protection at multiple other sites that are continuously exposed to commensal, pathogenic, and environmental microbes (22). The discovery that ICOSL functions as novel modulator of IL-22 responses will also open new avenues for the treatment of human inflammatory disorders including psoriasis, arthritis, and IBD.

## Supplementary Material

Data Supplement
