# Supplementary material for: Antigen-Presenting Human γδ T Cells Promote Intestinal CD4+ T Cell Expression of IL-22 and Mucosal Release of Calprotectin
Source: J Immunol. 2017 Mar 22;198(9):3417–25. doi: 10.4049/jimmunol.1700003 (PMC5392732; doi:10.4049/jimmunol.1700003)
Supplement: Data Supplement [file JI_1700003.zip › JI_1700003_Supplemental_Figures_1.pdf]

Antigen-presenting human γδ T-cells promote intestinal $CD4^+$ T-cell expression of IL-22 and mucosal release of calprotectin

Christopher J. Tyler[*], Neil E. McCarthy[†], James O. Lindsay[†,‡], Andrew J. Stagg[†], Bernhard Moser[*,§], and Matthias Eberl[*,§]

[*]*Division of Infection and Immunity, School of Medicine, Cardiff University, Cardiff CF14 4XN, United Kingdom;* [†]*Centre for Immunobiology, The Blizard Institute, Barts and The London School of Medicine and Dentistry, Queen Mary University of London (QMUL), London E1 2AT, United Kingdom;* [‡]*Department of Gastroenterology, The Royal London Hospital, Barts Health NHS Trust, London E1 1BB, United Kingdom;* [§]*Systems Immunity Research Institute, Cardiff University, Cardiff CF14 4XN, United Kingdom*

SUPPLEMENTAL DATA

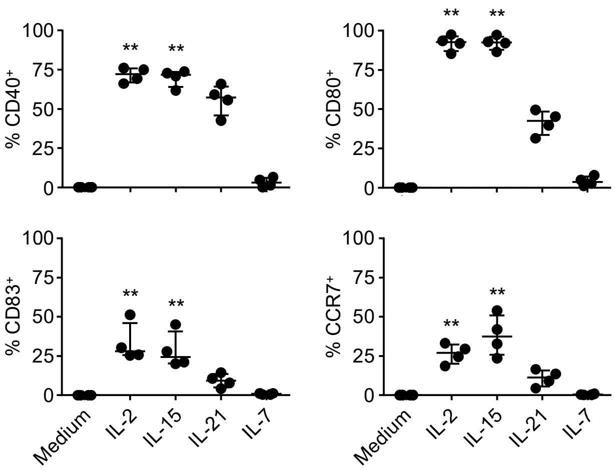

Supplemental Figure 1. Cytokine-dependent expression of APC markers by Vγ9/Vδ2 T-cells. Expression of CD40, CD80, CD83 and CCR7 by freshly isolated Vγ9/Vδ2 T-cells and by γδ T-APCs generated over three days in the presence of HMB-PP with the indicated cytokines, as gated on live single $V\gamma9^{+}$ T-cells. Data were analysed using Kruskal-Wallis tests combined with Dunn's multiple comparisons tests versus freshly isolated cells. Each data point represents an individual donor; asterisks depict significant differences. Horizontal lines display the median, error bars indicate the interquartile range.

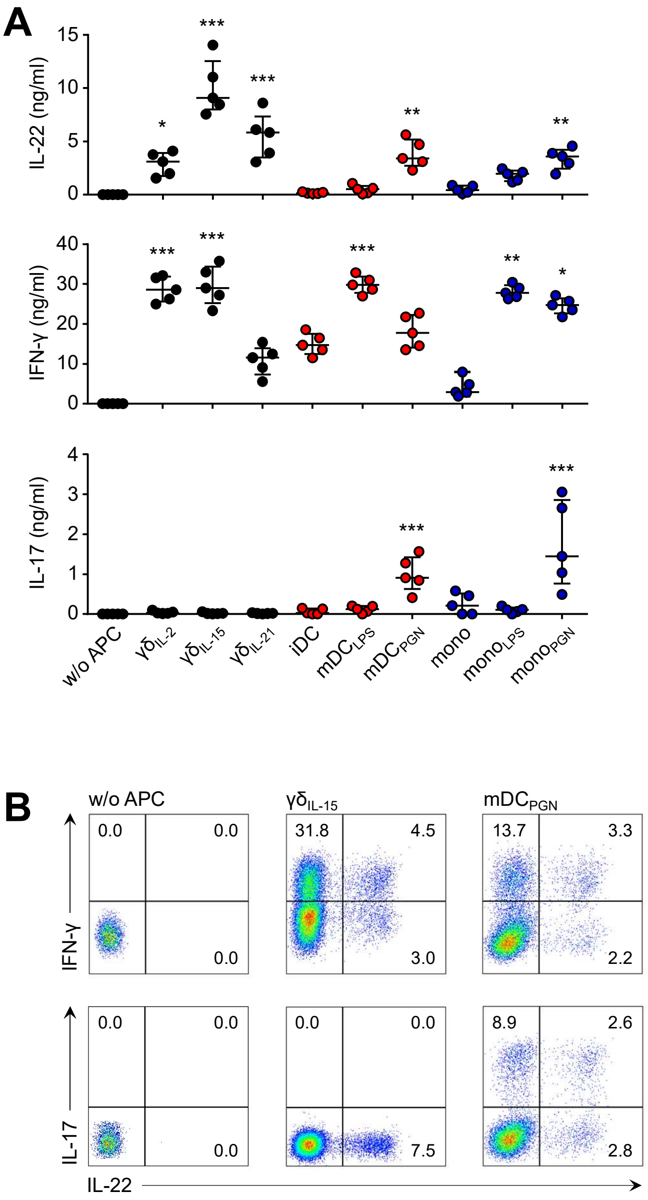

Supplemental Figure 2. APC-dependent polarisation of naïve CD4$^+$ T-cells. Naïve CD4$^+$ T-cells were co-cultured with γδ T-APCs generated under different conditions, in comparison with immature DCs (iDC), LPS or PGN-matured DCs (mDC_{LPS}, mDC_{PGN}), freshly isolated monocytes (mono), and LPS or PGN stimulated monocytes ($mono_{LPS}$, $mono_{PGN}$) from mismatched donors at an APC:responder ratio of 1:10; CD4$^+$ T-cells cultured alone (w/o APC) served as controls. (***A***) Cytokine secretion as assessed by ELISA after nine days in culture upon restimulation with PMA/ionomycin for 24 hours. (***B***) Cytokine profile of naïve CD4$^+$ T-cells stimulated by allogeneic $γδ_{IL-15}$ T-APCs as determined after nine days upon restimulation, compared to mDC_{PGN}. Data were analysed using Kruskal-Wallis tests combined with Dunn's multiple comparisons tests versus controls without APCs. Each data point represents an individual donor; asterisks depict significant differences. Horizontal lines display the median, error bars indicate the interquartile range. FACS plots are representative of at least four experiments using cells from at least four individual donors.

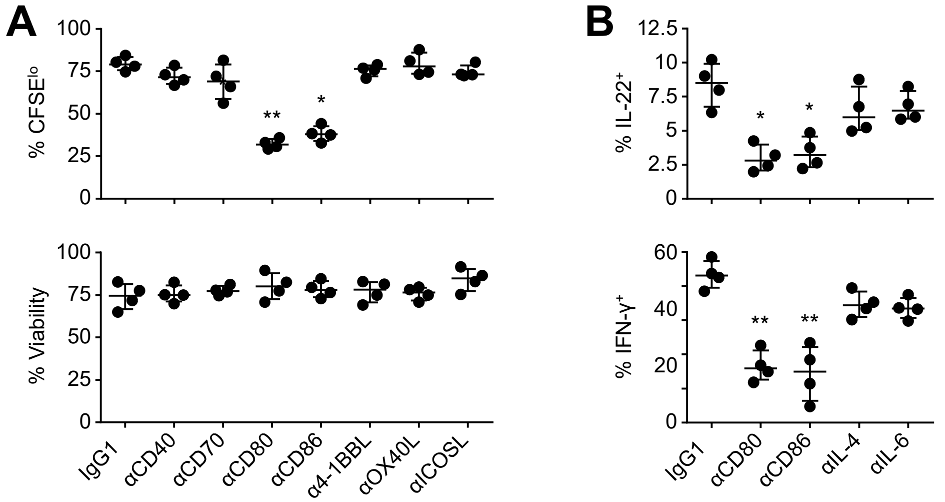

Supplemental Figure 3. Effect of co-stimulatory interactions on proliferation, viability and cytokine profiles of naïve CD4$^+$ T-cells. (***A***) CFSE-labelled naïve CD4$^+$ T-cells were co-cultured with $\gamma\delta_{IL\text{-}15}$ T-APCs from an allogeneic donor at an APC:responder ratio of 1:10. CD4$^+$ T-cell proliferation in the absence or presence of blocking antibodies was assessed by flow cytometry after five days and is displayed as percentage of CFSE^{lo} cells. Viability was assessed by flow cytometry after nine days and displayed as percentage of CD4$^+$ T-cells negative for dead cell staining. (***B***) Intracellular cytokine expression by naïve CD4$^+$ T-cells in response to allogeneic $\gamma\delta_{IL\text{-}15}$ T-APCs co-cultured in the absence or presence of blocking antibodies, as determined by flow cytometry after nine days upon restimulation with PMA/ionomycin for 5 hours. Data were analysed using Kruskal-Wallis tests combined with Dunn's multiple comparisons tests versus IgG1 controls. Each data point represents an individual donor; asterisks depict significant differences. Horizontal lines display the median, error bars indicate the interquartile range.

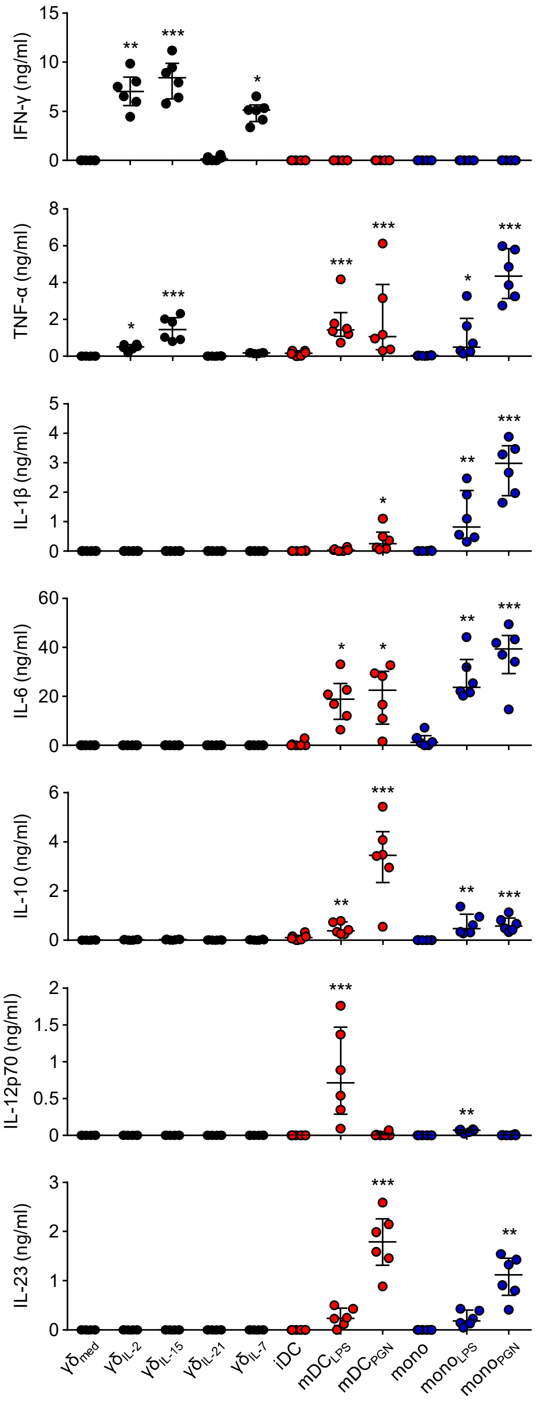

Supplemental Figure 4. Cytokine profile of distinct APC populations. Purified $V\gamma9/V\delta2$ T-cells were cultured in medium or stimulated with 10 nM HMB-PP alone and different common γ-chain cytokines for 24 hours. Immature DCs (iDC) and freshly isolated monocytes (mono) cultured in medium only or stimulated overnight with LPS or PGN served as controls. Cytokine levels in the culture supernatants were determined by ELISA. Data were analysed using Kruskal-Wallis tests combined with Dunn's multiple comparisons tests versus $\gamma\delta_{med}$ controls. Each data point represents an individual donor; asterisks depict significant differences. Horizontal lines display the median, error bars indicate the interquartile range.
